# Development and Psychometric Validation of Perioperative Patient-Reported Experience Measures for Nursing Care in Surgical Patients

**DOI:** 10.1155/jonm/3790942

**Published:** 2025-09-11

**Authors:** Chengyao Guo, Xiao Chen, Yi Wang, Zhe Wang, Qiaoran Li, Chunling Wang, Yuxia Zhang

**Affiliations:** Department of Nursing, Zhongshan Hospital, Fudan University, Shanghai, China

**Keywords:** patient-reported experience measures, perioperative nursing experience, reliability, surgical patients, validity

## Abstract

**Background:** Patient experience is a key indicator of healthcare quality. However, existing patient-reported experience measures (PREMs) predominantly focus on outpatient or general inpatient settings, with limited attention given to the perioperative period.

**Objective:** This study aimed to develop and validate a perioperative PREMs tool tailored to assess nursing care in surgical patients.

**Methods:** Adopting a mixed-methods approach guided by The International Association for Health Professions Education (AMEE) Guide No. 87, this study involved a literature review, semistructured interviews, expert consultations, cognitive interviews, pilot testing, and psychometric evaluation. Data were collected from 444 surgical patients at a tertiary hospital in China. Reliability (assessed through internal consistency and test–retest reliability) and validity (evaluated in terms of structural and content aspects) were assessed using Cronbach's α, intraclass correlation coefficient (ICC), confirmatory factor analysis (CFA), and factor analysis.

**Results:** The final 18-item PREMs tool comprised three dimensions: Environmental and Nursing Care (eight items), Emotional Support and Communication (seven items), and Information Support (three items). The total scale demonstrated excellent internal consistency (Cronbach's α = 0.942), with subscale α values all exceeding 0.8. Test–retest reliability was strong (ICC > 0.7), and CFA results indicated a good model fit (comparative fit index = 0.928 and root mean square error of approximation = 0.046), confirming robust psychometric properties.

**Conclusion:** The developed perioperative PREMs tool exhibited strong reliability and validity, offering a standardised means of evaluating surgical patients' experiences about nursing care and serving as a valuable resource for guiding targeted quality improvement initiatives.

## 1. Introduction

Patient experience is recognised as one of the key quality assessment criteria for healthcare institutions, as established by the Joint Commission International [1–3]. Distinct from patient satisfaction, patient experience refers to patients' actual encounters, perceptions, and interactions during clinical care [4]. A typical example of a patient experience question is “Did a nurse greet you upon your arrival for admission?” This type of measurement typically assesses the frequency of specific behaviours, using response options such as “Sometimes” or “Always” [5]. In contrast, patient satisfaction reflects the degree to which received services align with the patient's subjective expectations [6]. A representative satisfaction question might be “How satisfied are you with the nurse's attitude?” This construct is commonly measured using rating scales—often on a scale from 1 to 10 (where 1 = extremely dissatisfied and 10 = extremely satisfied) [7]. Patient satisfaction is considered an outcome variable, whereas patient experience is understood as a process variable, offering a tangible expression of the principles of patient-centred care [2].

Numerous countries have prioritised the measurement and improvement of patient experience as a crucial healthcare initiative [5]. In contrast to qualitative approaches, which often have limited capacity for data collection, quantitative instruments such as standardised scales and questionnaires enable the systematic collection and analysis of patient experience data [8]. Patient-reported experience measures (PREMs) are standardised assessment tools specifically designed to capture patients' first-hand perspectives regarding actual care encounters during treatment; rather than evaluating the experiences themselves, PREMs focus on documenting experiential facts [9–11]. This approach not only generates quantitative metrics but also provides detailed qualitative insights into the nature of patient experience [12]. PREMs have been widely adopted by the international healthcare community owing to three primary advantages: they comprehensively reflect diverse care experiences [13]; they establish a universal measurement framework that supports policymaking and healthcare reform [14]; and they provide patient-level, actionable data that can be used to enhance service quality [15].

However, existing PREMs implementations have predominantly focused on inpatient ward or outpatient experiences, with limited attention given to the intraoperative experiences of surgical patients [12]. As surgical intervention often represents the central component of inpatient care for this patient population, the intraoperative phase constitutes a pivotal aspect of their overall hospitalisation journey. Gaining a deeper understanding of patients' multifaceted experiences during this critical phase provides valuable insights for optimising their overall care experience. Furthermore, when patients choose to undergo surgery, their expectations often extend beyond the resolution of disease—they expect a complete alleviation of all associated discomfort. This paradigm shift in expectations necessitates a dual focus: achieving full functional recovery and simultaneously identifying the external determinants that contribute to patient experience [16]. Given that nursing care has been identified as the most important independent factor influencing patients' overall care experiences [17, 18], developing validated instruments to evaluate procedural nursing care experiences in surgical patients is essential for improving health outcomes. Therefore, this study aimed to develop a PREMs assessment tool tailored for assessing nursing care in perioperative settings, while also establishing a standardised assessment framework for systematically capturing and analysing real-time surgical nursing care experiences. This approach enables comprehensive quality evaluation while identifying modifiable factors for optimising perioperative nursing care.

## 2. Materials and Methods

### 2.1. Study Design

**Figure 1:**
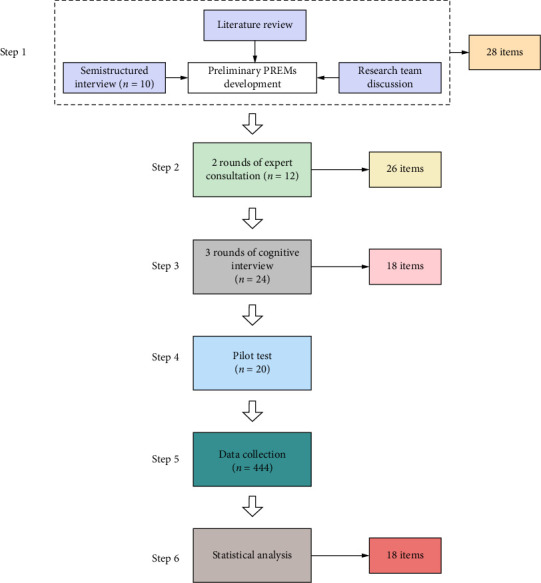
Systematic development workflow of perioperative PREMs.

The development of perioperative PREMs in this study followed the standardised framework outlined in AMEE Guide No. 87 [19]. This study was conducted at Zhongshan Hospital, Fudan University, between 2023 and 2025, in strict compliance with international research guidelines and quality assurance protocols [20, 21]. A systematic development workflow of the perioperative PREMs is presented in Figure 1. Ethical approval was obtained from the Institutional Review Board of Zhongshan Hospital, Fudan University. All participating researchers completed rigorous protocol training and signed informed consent forms that outlined the study's objectives, with assurances of their right to withdraw without prejudice.

### 2.2. Total Patients

A total of 498 surgical patients participated in this study. Of these, 10 were involved in semistructured interviews, 24 participated in cognitive interviews, and 20 took part in the pilot test. Subsequently, 444 patients contributed data for reliability and validity testing, including 29 who completed the test–retest assessment. The sampling details for each step are described below.

#### 2.2.1. Step 1: Preliminary Development of Perioperative PREMs

Between September and December 2023, a comprehensive literature search was conducted across PubMed, Web of Science, EMBASE, and CINAHL using the keywords “experience OR satisfaction” AND “operation OR surgery” to identify studies reporting on perioperative patient experiences. The extracted data were used to develop a preliminary pool of items. Existing PREMs relevant to surgical settings were critically evaluated to guide the scale design.

Postoperative patients in Zhongshan Hospital, Fudan University, were recruited for semistructured interviews between January and April 2024 using purposive sampling with a maximum variation strategy. The inclusion criteria were as follows: requirement of surgical elective procedures, postoperative status, and provision of informed consent. The exclusion criteria included language or communication barriers, cognitive impairment, and age under 18 years. The interviews were conducted until thematic saturation was achieved [22], resulting in a final cohort of 10 patients.

Interviews were conducted 1–2 days postoperatively (depending on each patient's postoperative conditions) to ensure that the patients were fully conscious and able to recall the surgical process clearly. The interviews specifically encouraged patients to describe their nursing care-related experiences. The interview protocol covered four domains.

• Treatment context: “What condition led to your hospitalisation?”
• Preoperative experience: “Describe your physical/emotional experiences while awaiting surgery.”
• Immediate postoperative experience: “What occurred, and how did you feel in the operating room before transfer to recovery/ICU?”
• Unmet needs: “What were your primary needs during the surgical process?”

All interviews were audio-recorded and transcribed verbatim. Data were analysed using NVivo 12 software, and Colaizzi's seven-step phenomenological analysis method was applied to code, categorise, and integrate emerging themes into an item pool.

A multidisciplinary research team synthesised the findings from the literature and conducted qualitative interviews through iterative discussions. This process yielded the first draft of the perioperative PREMs, ensuring alignment with both clinical realities and relevant theoretical frameworks.

#### 2.2.2. Step 2: Expert Delphi Consultation

Between May and July 2024, the preliminary versions of the perioperative PREMs were subjected to a Delphi expert consultation process. Experts were selected nationwide based on authority and representativeness. The inclusion criteria were ≥ 10 years of experience in nursing, surgery or anaesthesia; a professional title of intermediate level or higher, with either a master's degree or a bachelor's degree combined with ≥ 20 years of clinical experience; and a willingness to participate in the consultation. Exclusion criteria included unavailability owing to leave or training commitments and retirement or formal resignation.

The consultation materials comprised the following components:

• Introduction: an overview of the research background, objectives and key definitions.
• Draft PREMs: a set of preliminary scale items for evaluation.
• Scoring system: a five-point Likert scale (ranging from 5 = fully appropriate to 1 = completely inappropriate), assessing item relevance to surgical experiences. Each item included open fields for expert feedback.
• Expert profile survey: demographic and professional information including sex, age, education, work experience, professional title and specialty.
• Expertise evaluation: a self-assessment of familiarity with perioperative care, along with the basis for their professional judgment (e.g., theoretical knowledge and clinical experience).

The research team conducted two rounds of Delphi consultation via email. To maintain rigour, experts were required to submit their responses within 10 days. Questionnaire data were processed systemically using the following dual evaluation criteria.

• Objective thresholds: items were retained if they achieved a mean importance score of > 3.5 and coefficient of variation of < 0.25 [23].
• Subjective analysis: qualitative feedback from experts was thematically synthesised to guide item revisions.

Revised questionnaires were redistributed at one-month intervals until consensus was reached. After two iterative rounds, the multidisciplinary team revised and improved the preliminary version of the PREMs, incorporating evidence-based modifications based on expert consensus.

#### 2.2.3. Step 3: Cognitive Interviews

To refine the preliminary perioperative PREMs into testable versions, cognitive interviews were conducted with surgical patients between August and October 2024. Patients were recruited using the same inclusion and exclusion criteria as those applied during the semistructured interviews. Following methodological recommendations, three iterative rounds of interviews were conducted, involving 5–15 patients per round [24]. To ensure interview quality, each patient was interviewed with no more than 35 questions. The interview protocol covered the following areas:

1. General Comprehension:
  • “Were any scale items unclear? If so, which ones, and why?”
  • “Did any items seem irrelevant to your experience? Specify and explain.”
  • “Did any items cause discomfort or seem inappropriate? Elaborate.”
  • “How would you rephrase problematic items?”
  • “Suggest additions or deletions to the scale.”
2. Instructions:
    “Was the guidance text clear? Paraphrase it in your own words.”
3. Item-Specific Probing:
  • Paraphrasing: “Restate this item in your own terms.”
  • Interpretation: “How do you understand this item?”
  • Confidence: “How certain are you about your response?”
  • Recall: “What experiences influenced your answers?”
  • Keyword Clarification: “What does [specific term] mean to you?”
  • Usability: “Was this item easy/difficult to answer? Any wording concerns?”

Prior to the interviews, patients were provided with detailed explanations of the study's objectives and procedures, after which they gave written informed consent. The methodology combined the think-aloud technique with structured probing. Patients were first asked to verbalise their cognitive processes while completing the scale, followed by systematic questioning aligned with the content of the perioperative PREMs. Subsequently, audio recordings and field notes were transcribed verbatim within 24 h and subjected to thematic analysis. The research team revised the PREMs based on the interview findings and initiated further rounds of interviews until data saturation was achieved (i.e., no new themes emerged across three consecutive interviews).

#### 2.2.4. Step 4: Pilot Testing

Prior to formal validation, a pilot test was conducted in November 2024 at Zhongshan Hospital, Fudan University, using convenience sampling to recruit 20 surgical patients [23]. The same inclusion and exclusion criteria as those used in the semistructured interviews were applied. The primary aim of the pilot survey was to validate the scale's content accuracy, readability and ambiguity by collecting real-time feedback on item clarity, relevance and response burden. Patients completed the pilot version of the PREMs, while researchers documented usability issues, misinterpretations and completion time [23, 25].

#### 2.2.5. Step 5: Data Collection

Between December 2024 and March 2025, convenience sampling was used to recruit surgical patients at Zhongshan Hospital, Fudan University, in accordance with predefined inclusion and exclusion criteria. Based on psychometric standards requiring a sample size five to ten times the number of questionnaire items [26], a minimum of 370 patients was required. To account for potential attrition, an additional 10% was added, bringing the total target sample to 407. The inclusion criteria were as follows: requirement of elective surgical procedures, postoperative status and provision of informed consent. The exclusion criteria included language or communication barriers, cognitive impairment and age under 18 years. Recruitment posters were displayed in various surgical wards, and eligible patients were invited to participate. Patients who expressed interest were given an invitation letter outlining the study's purpose, procedures and ethical approval, as well as the informed consent form. Upon formal agreement to take part in the study, written informed consent was obtained from each patient, after which data collection commenced.

Data collection was conducted in hospital wards by trained nurses, who explained the questionnaire content to patients 1–3 days after surgery. Paper-based questionnaires were distributed, comprising three sections: demographic data, including age, sex, occupation, education level, residence and income; disease-related information, such as previous surgery, surgery type, surgery duration, sequential surgery and scheduling time; and the test version of the perioperative PREMs. Completed questionnaires were collected and reviewed by nurses.

Additionally, electronic questionnaires were distributed to a subgroup of 31 patients via WeChat or email two weeks after preliminary completion to assess test–retest reliability [27]. To ensure sample representativeness, patients were selected using a maximum variation sampling strategy. Furthermore, it was confirmed that none of the patients had experienced further healthcare encounters or other significant life events during the 2-week interval between measurements, to minimise potential influences on the reliability of the results.

#### 2.2.6. Step 6: Statistical Analysis

##### 2.2.6.1. Reliability Testing

Internal consistency: Evaluated using Cronbach's α coefficient (α ≥ 0.7, acceptable; α ≥ 0.8, good; α ≥ 0.9, excellent) [23].

Item discrimination: Assessed using the separation statistic (values ≥ 5 indicates good discrimination) [28].

Test–Retest reliability: Stability was tested using the intraclass correlation coefficient (ICC), with ICC > 0.7 indicating high consistency [27].

Factor analysis appropriateness: Determined using the Kaiser–Meyer–Olkin (KMO) measure (≥ 0.6, acceptable; ≥ 0.8, excellent) and Bartlett's test of sphericity (*p* < 0.05) [23].

Validity testing: Confirmatory factor analysis (CFA) was conducted to assess structural validity. The model fit was assessed using the following indices: comparative fit index (CFI ≥ 0.90), Tucker–Lewis index (TLI ≥ 0.90), root mean square error of approximation (RMSEA < 0.08) and standardised root mean square residual (SRMR < 0.08) [29].

Data were double-entered into Excel and analysed using IBM SPSS 25.0 and AMOS 27.0. Results with *p* < 0.05 were considered statistically significant.

## 3. Results

Based on the literature review and findings from qualitative interviews, the research team developed a preliminary version of the perioperative PREMs comprising 28 items, which were refined through group discussions. The scale adopted a five-point Likert scoring system, ranging from “Never/Strongly Disagree” to “Always/Strongly Agree.”

Twelve experts participated in two rounds of Delphi consultation. The expert panel had a mean age of 49.1 ± 14.6 years and an average of 28.8 ± 3 years of professional experience. Details of the expert panel are presented in Table 1. After evaluation, 26 items were retained.

A total of 24 surgical patients participated in cognitive interviews conducted across three phases: 12 patients in the first round, eight in the second round and four in the final round. The cohort comprised 16 males and eight females, with a mean age of 55 ± 15.28 years. Educational backgrounds were as follows: junior high school or below (*n* = 13), senior high school or vocational secondary education (*n* = 3), college diploma or bachelor's degree (*n* = 6) and master's degree or higher (*n* = 2). All patients were insured under the National Health Insurance System, with 50% (*n* = 12) reporting previous surgical experience. The surgical procedures included open surgery under general anaesthesia (*n* = 9), laparoscopic surgery under general anaesthesia (*n* = 7), procedures under local anaesthesia (*n* = 4) and interventional radiological procedures (*n* = 4). In the first two rounds, patients expressed objections to certain items, and their feedback, along with subsequent revisions, is detailed in Tables 2 and 3. No objections to the items were raised during the third round of interview. Based on iterative revisions informed by the interview findings, the research team finalised an 18-item pilot version of the PREMs.

During the pilot test step, 20 surgical patients from Zhongshan Hospital, Fudan University—including 11 males and nine females aged between 20 and 80 years—participated in the study. Based on findings from this preliminary evaluation, the research team refined the measurement instrument by addressing issues related to questionnaire distribution and completion processes.

The finalised 18-item PREMs used for reliability and validity testing comprised three dimensions: Dimension 1—Environment and Nursing Services (Q1–Q8), Dimension 2—Emotional Support and Communication (Q9–Q15) and Dimension 3—Information Support (Q16–Q18). Patient experiences were measured using a five-point Likert scale (1 = strongly disagree/never/not at all; 5 = strongly agree/always/completely), with “not applicable” responses excluded from scoring. Higher scores indicated better perioperative experiences.

### 3.1. Patient Characteristics for Reliability and Validity Testing

A total of 550 questionnaires were distributed, with 498 returned, yielding a response rate of 90.5%. After excluding incomplete or patterned responses, 444 valid questionnaires were retained (effective rate: 89.2%). The patients' demographic characteristics are presented in Table 4.

### 3.2. Reliability Testing

#### 3.2.1. Internal Consistency and Discrimination

The questionnaire demonstrated excellent internal consistency (Cronbach's α = 0.942). All subscales achieved α values exceeding 0.8 (Table 5). Separation indices ranged from 14.569 to 18.340 (all > 5), indicating strong item discrimination.

### 3.3. Test–Retest Reliability

Among the 31 patients included in the test–retest reliability assessment, 29 provided valid retest data after a 2-week interval. The ICC values for the total questionnaire and subscales exceeded 0.7, confirming stability (Table 6).

### 3.4. Validity Analysis

#### 3.4.1. Factor Analysis Appropriateness

The KMO measure was 0.93, and Bartlett's test of sphericity yielded *χ*^2^ = 8733.766 (df = 300, *p* < 0.001), confirming that the data were suitable for factor analysis (Table 7).

### 3.5. Structural Validity

**Figure 2:**
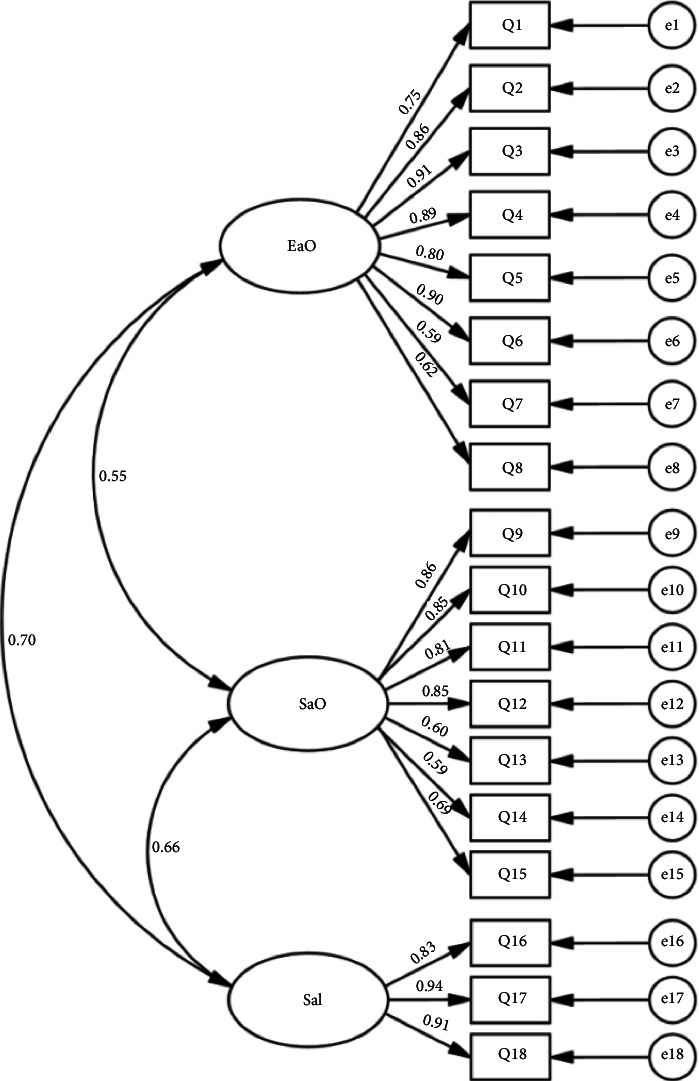
Confirmatory factor analysis path diagram.

The CFA demonstrated that all factor loadings exceeded 0.3 (Figure 2). Model fit indices were *χ*^2^/df = 1.943 (< 5), CFI = 0.928, TLI = 0.917, RMSEA = 0.046 (< 0.05) and SRMR = 0.083 (approaching 0.08), indicating good model fit (Table 8).

The analysis demonstrated that the 18-item PREMs exhibit good reliability and validity. The 18-item Perioperative PREMs for Nursing Care in Surgical Patients are illustrated in Supporting file 1.

## 4. Discussion

Effectively capturing patients' authentic voices and lived experiences has long been a key priority in improving patient-centred care, hence the emphasis placed on the development and application of PREMs in this study. Unlike satisfaction surveys, which subjectively assess the extent to which patients' expectations are met, PREMs focus on capturing specific, detailed accounts of patients' actual experiences [30]. Regarding the scope of PREMs, systematic reviews of existing tools have demonstrated that these measures comprehensively cover key elements of the patient's experience [31]. Typically administered as structured questionnaires [32], PREMs have been widely adapted for use across diverse clinical settings, including surgery, chronic disease management, paediatrics, emergency care and oncology [9, 14, 33, 34]. As standardised measurement tools, PREMs also require rigorous psychometric validation prior to clinical implementation. Systematic reviews have confirmed the robust reliability and validity of existing PREMs [5, 15], underscoring the methodological rigour and completeness of their development. However, despite their structured design, studies have indicated that PREMs remain relatively underrecognised and underutilised compared with the well-established Patient-Reported Outcome Measures [30]. This underscores the importance of further expanding the development of standardised PREMs to strengthen systems for evaluating patient experience.

The perioperative experience forms a pivotal component of a patient's overall hospital stay and directly influences the perception of care quality. However, most studies on surgical patient experiences have focused on the preoperative phase, rather than encompassing the entire perioperative period. For example, research on patients undergoing rotator cuff repair has explored how preoperative expectations and concerns affect postoperative experience [16]. Similarly, a review of studies on orthopaedic surgical experiences revealed that the existing literature predominantly investigated preoperative expectations and their effect on postoperative satisfaction, with few tools specifically designed to measure the full perioperative experience [35]. Additionally, many studies continue to rely on satisfaction surveys, such as those evaluating whether early postoperative bathing improves satisfaction [36] or assessing the effect of informed decision-making on satisfaction with surgical outcomes [37]. Collectively, these findings suggest a growing consensus that perioperative experiences play a vital role in shaping patient perceptions [35], necessitating more scientifically robust measurement systems to objectively capture these experiences.

The development of perioperative PREMs for nursing care in surgical patients was grounded in authentic patient narratives that recognise patients as experts in their own health journeys. Therefore, ensuring that patients' voices guide the development process of PREMs is essential [38, 39]. To construct a preliminary item pool, we complemented the literature review with semistructured interviews with postoperative patients, extracting critical themes from their experiences. For example, patients raised concerns such as *“The operating room was too cold—could the temperature be adjusted beforehand?”*; “*I couldn't distinguish roles among staff—who was the anesthesiologist versus the nurse? A brief introduction would help.”*; and *“I felt disoriented during the process, unsure of where I was being moved or what would happen next.”* During the cognitive interviews, patients provided constructive feedback on the draft PREMs. For instance, patients critiqued *“Item 3: The operating theatre nursing staff maintained a neat and pretty appearance,”* describing it as ambiguous. They pointed out that *“patients cannot judge ‘appearance' but can assess whether attire complies with protocols.”* Similarly, in response to *“Item 7: Did nursing staff demonstrate procedural competence during clinical procedures?”* patients argued that *“procedural competence should be evaluated by supervisors, not patients, who can only assess whether actions were performed carefully and skillfully.”* Others also observed that the initial 26-item PREMs were burdensome during postoperative recovery, with redundant items warranting consolidation. It is widely acknowledged that instruments containing fewer items are more likely to be completed by patients, thereby increasing the likelihood of obtaining high-quality responses [40]. Through iterative revisions, the research team refined the tool into an 18-item pilot version for psychometric testing.

The perioperative PREM for nursing care in surgical patients comprised three dimensions: environmental and nursing services, emotional support and communication and information support. These dimensions align with established indicators for enhancing patient experience. For example, Jones' et al. PREMs-based study of emergency surgical patients identified strong correlations between positive experiences and effective communication, information support and quieter environments [33]. Moreover, there is a consensus that PREMs should encompass domains such as communication, information provision, care, physical/psychological support, shared decision-making and environmental quality [31, 38]. This study achieved an adequate number of valid questionnaire responses, based on the calculated sample size, to ensure analytical robustness. Demographic variables (age, sex, education, occupation and income) and surgical characteristics (procedure type and anaesthesia method) were evenly distributed to minimise sampling bias. The final psychometric tests confirmed the strong reliability and validity of the PREMs.

The development of PREMs requires a structured process involving model establishment, patient-driven content generation, hierarchical validation and psychometric evaluation [41, 42]. This study, guided by the AMEE framework, followed a rigorous scale development process, encompassing item pool construction, expert consultation, cognitive interviews, pilot testing and formal validation. A series of PREMs have been developed to assess the hospitalisation experiences of adult inpatients. A systematic review examined the development processes and psychometric property testing of relevant PREMs [5], identifying approximately 23 existing PREMs for evaluating inpatient experiences. Of these, nine studies either omitted or failed to report pilot testing and cognitive interviews, and only five assessed content validity. Among the assessed measurement properties, internal consistency and structural validity were the two most frequently tested characteristics. Notably, only a small proportion of existing PREMs measuring adult inpatient experience were developed and validated using methodologically rigorous approaches. In contrast, the PREMs developed in the present study followed a comprehensive and systemic approach. Although indicators of content validity were not explicitly tested as part of the psychometric validation phase, high content validity was ensured through expert consultation.

This study had some limitations. Although the research team maximised patient diversity during semistructured interviews, cognitive interviews and psychometric testing, this study was conducted at a single hospital, limiting the generalisability of the findings to broader populations. Additionally, time constraints precluded comprehensive validation of all psychometric properties (e.g., content validity). Future research will aim to recruit patients from a wider geographical spread across the country for large-scale, multi-centre studies to further validate the applicability of the PREMs. First, stratified sampling will be employed based on geographical location (e.g., eastern, central and western regions) and hospital tier (tertiary/primary care hospitals) to ensure representative sampling across regions with varying levels of healthcare resources, thereby enhancing generalisability. Simultaneously, a hybrid approach combining offline hospital recruitment with online platform deployment will be adopted to proactively diversify sampling sources. Second, standardised training will be provided to researchers at all participating centres to minimise operational variations. To further promote the dissemination of the established PREMs, future efforts will include proactive collaboration with international experts to conduct cross-cultural adaptation—including translation and linguistic validation—for localised implementation tailored to diverse cultural and linguistic contexts.

## 5. Conclusions

This study successfully developed and validated a psychometrically sound PREMs tool tailored to assess nursing care in surgical patients during the perioperative period. The final instrument comprised 18 items organised into three dimensions, utilising a 5-point Likert scale to comprehensively evaluate patients' nursing care experiences and perceptions throughout their surgical journey. As a standardised and objective measurement tool, the perioperative PREMs provide a systematic framework for assessing key elements of patient-centred care and offer actionable insights for improving the quality of surgical experiences. Moreover, its robust reliability and validity underscore its potential utility in both clinical practice and research to advance patient experience optimisation initiatives.

## Figures and Tables

**Table 1:** Expert panel information (*n* = 12).

Expert ID	Academic degree	Academic title	Research focus	Institutional affiliation
E-01	Bachelor	Associate senior	Anaesthesia nursing	The First Affiliated Hospital, Sun Yat-Sen University
E-02	Bachelor	Senior practitioner	Patient experience research	Peking Union Medical College Hospital
E-03	Master	Associate senior	Nursing management	Zhongshan Hospital, Fudan University
E-04	Bachelor	Senior practitioner	Anaesthesia nursing	Shengjing Hospital of China Medical University
E-05	Master	Senior practitioner	Nursing management	Ruijin Hospital, Shanghai Jiao Tong University School of Medicine
E-06	Master	Associate senior	Nursing management	Sir Run Run Shaw Hospital, Zhejiang University School of Medicine
E-07	Bachelor	Senior practitioner	Operating room nursing	Xiangya Hospital of Central South University
E-08	Bachelor	Senior practitioner	Operating room nursing	West China Hospital, Sichuan University
E-09	Master	Associate senior	Nursing management	Affiliated Drum Tower Hospital, Medical School of Nanjing University
E-10	Doctor	Associate senior	Patient experience research	Zhongshan Hospital, Fudan University
E-11	Master	Associate senior	Operating room nursing	The First Affiliated Hospital of Zhengzhou University
E-12	Master	Associate senior	Patient experience research	Union Hospital, Tongji Medical College, Huazhong University of Science and Technology

**Table 2:** Patient feedback and item modifications from the first round of cognitive interviews.

Original items	Patient feedback	Item modifications	Modified items
Overall assessment of PREMs	① One patient (1-p6) remarked that the inclusion of temporal qualifiers (e.g., “during your admission”) across multiple items increased the cognitive load by requiring them to recall and verify the accuracy of time periods. This patient further noted that numerous items with similar content were distinguished only by time frames, suggesting the consolidation of such items to reduce respondent burden. ② Three patients (1-p5, 1-p8 and 1-p12) identified substantial item redundancy in the scale, recommending that semantically overlapping items be merged or removed.	Regarding the recommendation to remove temporal specifications, the expert panel concluded that deleting time qualifiers and consolidating identical items was methodologically justified. The consolidated item would assess patients' experiences throughout the surgical procedure, as response options were anchored to the frequency of occurrence. This approach permits holistic evaluation of the care continuum. Regarding item redundancy, the panel agreed that eliminating duplicative items would enhance the scale's discriminant validity, thereby streamlining the scale without compromising conceptual coverage.	Remove temporal qualifiers from the items; consolidate or eliminate semantically overlapping items.

R1-3. Operating theatre nursing staff maintained a neat and pretty appearance.	① Conceptual Ambiguity: Five patients (1-p2, 1-p3, 1-p4, 1-p5 and 1-p7) expressed concerns regarding the term “pretty appearance,” citing ambiguous evaluation criteria and explicitly asking “what constitutes the threshold for acceptable pretty appearance?” ② Content relevance: Three patients (1-p3, 1-p7 and 1-p11) questioned the item's clinical salience, stating that the appearance of nursing staff was a low-priority concern provided clinical dress code compliance was maintained.	Following panel deliberation, it was concluded that patients' primary concern regarding the appearance of nursing staff within the surgical context centred on adherence to professional dress codes. Consequently, the original descriptor “neat and pretty appearance” was revised to “adherence to professional dress codes.”	R2-3. Operating theatre nursing staff adhered to professional dress codes.

R1-4. Nursing staff provided detailed explanations of personnel, environment, surgical procedures, and relevant precautions during preoperative visits.	① Item interpretation: Four patients (1-p4, 1-p5, 1-p9 and 1-p10) contended that explanations of surgical process should be provided throughout the perioperative period, not solely during preoperative visits. They interpreted the item's wording as implying that such communication was exclusively required preoperatively, potentially neglecting intraoperative and postoperative education. ② Cultural context relevance: Five patients (1-p2, 1-p3, 1-p4, 1-p6 and 1-p10) emphasised familial inclusion in surgical communication, noting that within China's healthcare paradigm, (a) preoperative preparations often require family participation, and (b) intraoperative protocol modifications necessitate surgeon-family discussions to ensure informed comprehension.	Following panel deliberation, it was agreed that surgical process education should take place across all perioperative phases, with nurses providing phase-specific explanations. Consequently, temporal descriptors were eliminated to expand the item's applicability to the entire surgical journey, and the phase “to you/your family” was added.	R2-4. Nursing staff provided comprehensive explanations to you/your family regarding personnel, environment, surgical procedures and precautions.

R1-5. Did nursing staff provide psychological support and reassurance during your preoperative visit?	① Item redundancy: Five patients (1-p2, 1-p4, 1-p5, 1-p6 and 1-p7) identified substantial content overlap between these items and R1-20, noting that all aimed to assess nurses' caring behaviours towards patients. ② Presumptive condition bias: Two patients (1-p2 and 1-p12) raised concerns about an implicit assumption in the items: that preoperative anxiety was present and necessitated psychological intervention. They noted that this presumption excluded patients who felt calm and did not require such support, potentially introducing forced response bias.	Following panel deliberation, it was concluded that item R1-20—“did nursing staff proactively provide companionship and emotional support?”—adequately encompasses the constructs measured by both redundant items, while maintaining universal applicability across varying patient psychological states.	Delete two items.
R1-9. Did nursing staff provide psychological support and reassurance while awaiting surgery?			

R1-6. Upon entering the operating theatre, did the nurse staff promptly introduce themselves to you?	Item Redundancy: Three patients (1-p3, 1-p6, 1-p10) identified conceptual duplication between this item and R1-7 (“upon entering the operating theatre, did operating room nurses provide a patient reception including preoperative process orientation”). They noted that self-introduction constitutes an integral initial component of the reception process within routine clinical practice.	Following expert panel deliberation, the patient feedback was endorsed. Consequently, item R1-7 was retained, and the duplicate item was deleted.	Delete item

R1-10. Did nurse staff provide adequate informational support to alleviate anxiety while you awaited surgery?	① Conceptual ambiguity: Four patients (1-p4, 1-p6, 1-p10 and 1-p11) reported difficulty understanding the term “informational support,” requesting simplified phrasing. ② Item redundancy: Two patients (1-p5 and 1-p9) raised concerns about the item's inherent evaluation bias, noting that patients may lack a clear standard for judging “adequacy.” they emphasised that timely response to patient inquiries should suffice as an indicator of sufficient information provision.	Following expert panel deliberation, conceptual ambiguity in this item was confirmed. As corroborated by patient feedback, the content queried is sufficiently addressed by existing items: R1-19 (“were your questions addressed thoroughly and patiently by nursing staff?”) and R1-23 (“did nursing staff respond promptly when assistance was required?”).	Delete item

R1-12. Did nursing staff ensure you were aware of and comprehended their procedural actions during clinical procedures?	① Conceptual ambiguity: Five patients (1-p5, 1-p8, 1-p9, 1-p10 and 1-p12) reported difficulty understanding the term the term “clinical procedures.” Three patients (1-p6, 1-p8 and 1-p9) expressed that they were unable to evaluate “procedural competence” owing to a lack of professional expertise, and suggested reframing the item to focus on observable behaviours. ②: Content relevance concerns: Two patients (1-p6 and 1-p8) questioned R1-15's clinical salience, noting that certain interventions, such as venipuncture or electrode placement, inherently cause discomfort. They cautioned against misattributing inherent procedural discomfort to nursing quality and instead proposed measuring aspects such as procedural fluidity and attentiveness to prevent technique-related discomfort. ③: Item redundancy and burden: Three patients (1-p5, 1-p8, 1-p12) identified conceptual overlap between R1-12 and R1-13 and recommended consolidating them into a single item.	Following comprehensive team deliberation on patient feedback, the four items addressing clinical procedures were deemed excessive. To address issues of terminological ambiguity and questionable clinical relevance raised by patients, the research team conducted a literature review and referenced the inpatient nursing service experience questionnaire. Consequently, content related to procedural explanations, procedural competence, and discomfort experience was consolidated into a single item assessing the technical proficiency of clinical techniques.	R2-7. How would you rate the technical proficiency of nursing staff during clinical procedures?
R1-13. Did nursing staff explain clinical procedures to you while performing them?			
R1-7. Did nursing staff demonstrate procedural competence during clinical procedures?			
R1-15. Did any clinical procedures performed by nursing staff cause you to experience physical discomfort?			

R1-26. Did operating room nursing staff facilitate your active participation in postoperative rehabilitation activities?	Content relevance concerns: Five patients (1-p3, 1-p4, 1-p5, 1-p8 and 1-p10) emphasised that postoperative rehabilitation should be primarily managed by ward nurses. They noted that ward nurses possess a comprehensive understanding of patients' perioperative health status, making them optimally positioned to lead rehabilitation activities. While acknowledging potential involvement from operating room nurses, they advocated for a supporting rather than primary role in postoperative recovery guidance.	Following expert panel deliberation on patient feedback, the unique intraoperative insights of operating room nurses (e.g., blood loss dynamics, skin integrity status and intraoperative thermoregulation) were acknowledged as clinically significant to postoperative recovery. However, under China's accelerated recovery after surgery framework, postoperative rehabilitation remains primarily coordinated by ward nursing teams. Given that ward nurses systematically incorporate intraoperative vital metrics into recovery planning, the panel concluded that postoperative follow-up counselling by operating room nurses should focus specifically on surgery-specific health guidance, rather than providing general rehabilitation instruction.	R2-18. During postoperative follow-up, did nursing staff provide tailored postoperative care instructions?

*Note:* 1-Px = Patient *x* in Round 1 interviews, R1-*x* = Item *x* in Round 1 interviews, R2-*x* = Item *x* in Round 2 interviews.

**Table 3:** Patient feedback and item modifications from the second round of cognitive interviews.

Original items	Patient feedback	Item modifications	Modified items
R2-5. Upon entering the operating theatre, did operating room nurses provide a patient reception including preoperative process orientation?	Two patients (2-P5 and 2-P7) recommended consolidating the item, noting that “patient reception” inherently includes “preoperative process orientation,” thereby rendering the latter phrase redundant.	Following expert panel deliberation, the patient feedback was formally endorsed.	R3-5. Upon entering the operating theatre, were you promptly received by nursing staff?

*Note:* 2-Px = Patient *x* in Round 2 interviews, R2-*x* = Item *x* in Round 2 interviews, R3-*x* = Item *x* in Round 3 interviews.

**Table 4:** Demographic characteristics of patients for reliability and validity testing (*n* = 444).

Variable	Category	*N*	%
Sex	Male	254	57.21%
	Female	190	42.79%

Age	Median [Q1, Q3]	61 [50, 68]	

Occupation	Office worker/Civil servant	79	17.79%
	Self-employed	46	10.36%
	Farmer	64	14.41%
	Labourer	35	7.88%
	Unemployed	28	6.31%
	Retired	192	43.24%

Education	Junior high or below	210	47.30%
	Senior high/Technical school	102	22.97%
	College/Bachelor's degree	116	26.13%
	Master's or higher	16	3.60%

Residence	Rural	81	18.24%
	Suburban	112	25.23%
	Urban	251	56.53%

Monthly Household income (CNY)	< 3000	58	13.06%
	3000–6000	178	40.09%
	6001–10,000	154	34.68%
	> 10,000	54	12.16%

Previous surgery	None	214	48.20%
	1–2 times	215	48.42%
	≥ 3 times	15	3.38%

Surgery type	Open surgery (general anaesthesia)	129	29.05%
	Laparoscopic surgery (general anaesthesia)	290	65.32%
	Local anaesthesia	23	5.18%
	Interventional	2	0.45%

Surgery duration (h)	< 3	34	7.66%
	3–6	324	72.97%
	> 6	86	19.37%

Sequential surgery	Yes	331	74.55%
	No	113	25.45%

Scheduling time	7:00–10:00	163	36.71%
	11:00–14:00	141	31.76%
	15:00–18:00	117	26.35%
	After 19:00	23	5.18%

**Table 5:** Cronbach's alpha and separation indices.

Dimension	Items	Cronbach's α	Separation index
Environment and operational standards	8	0.924	14.569
Emotional support and communication	7	0.880	10.008
Service information Coherence	3	0.921	12.729
Total questionnaire	18	0.942	18.340

**Table 6:** Test–retest reliability (ICC) (*n* = 29).

Dimension	ICC (2, 1)	95% CI	*p*-value
Total questionnaire	0.87	[0.82, 0.91]	< 0.001
Environment and operational standards	0.73	[0.65, 0.80]	0.012
Emotional support and communication	0.82	[0.75, 0.88]	0.003
Service information Coherence	0.76	[0.69, 0.83]	0.008

**Table 7:** KMO and Bartlett's test results.

KMO		0.93

Bartlett's test	*χ* ^2^	8733.766
	df	300
	*p*	< 0.001

**Table 8:** CFA model fit indices.

Index	*χ* ^2^/df	CFI	TLI	RMSEA	SRMR
Value	1.943	0.928	0.917	0.046	0.083

## Data Availability

The data that support the findings of this study are available from the corresponding author upon reasonable request.

## Supporting Information

Supporting file 1: The 18-item Perioperative PREMs for Nursing Care in Surgical Patients.
