# Supplementary material for: Development and Psychometric Validation of Perioperative Patient-Reported Experience Measures for Nursing Care in Surgical Patients
Source: J Nurs Manag. 2025 Sep 11;2025:3790942. doi: 10.1155/jonm/3790942 (PMC12446594; doi:10.1155/jonm/3790942)
Supplement: Supporting Information — Additional supporting information can be found online in the Supporting Information section. [file 3790942.f1.docx]

Perioperative Patient-Reported Experience Measures for Nursing Care in Surgical Patients

**Environment and Nursing Services**

1. The operating theatre environment was free from noticeable noise with adequate lighting.

(1) Strongly Disagree (2) Disagree (3) Neutral (4) Agree (5) Strongly Agree

2. The operating theatre maintained appropriate temperature, humidity, and air quality.

(1) Strongly Disagree (2) Disagree (3) Neutral (4) Agree (5) Strongly Agree

3. Operating theatre nursing staff adhered to professional dress codes.

(1) Strongly Disagree (2) Disagree (3) Neutral (4) Agree (5) Strongly Agree

4. Nursing staff provided comprehensive explanations to you/your family regarding personnel, environment, surgical procedures, and precautions.

(1) Strongly Disagree (2) Disagree (3) Neutral (4) Agree (5) Strongly Agree

5. Upon entering the operating theatre, were you promptly received by nursing staff?

(1) Strongly Disagree (2) Disagree (3) Neutral (4) Agree (5) Strongly Agree

6. Did nursing staff thoroughly verify your identity (name, hospital ID, bed number) upon arrival?

(1) Strongly Disagree (2) Disagree (3) Neutral (4) Agree (5) Strongly Agree

7. How would you rate the technical proficiency of nursing staff during clinical procedures?

(1) Not at all (2) Rarely (3) Occasionally (4) Mostly (5) Completely

8. Were you treated with respect and courtesy by nursing staff?

(1) Not at all (2) Rarely (3) Occasionally (4) Mostly (5) Completely

**Emotional Support and Communication**

9. When experiencing discomfort, did nursing staff respond promptly and attempt to alleviate it?

(1) Never (2) Seldom (3) Sometimes (4) Frequently (5) Always (6) Not applicable

10. Did nursing staff proactively provide companionship and emotional support?

(1) Not at all (2) Rarely (3) Occasionally (4) Mostly (5) Completely

11. Did nursing staff offer reassurance/encouragement when you experienced anxiety, fear, or distress?

(1) Never (2) Seldom (3) Sometimes (4) Frequently (5) Always (6) Not applicable

12. Were your questions addressed thoroughly and patiently by nursing staff?

(1) Never (2) Seldom (3) Sometimes (4) Frequently (5) Always (6) Not applicable

13. Did nursing staff respond promptly when assistance was required?

(1) Never (2) Seldom (3) Sometimes (4) Frequently (5) Always (6) Not applicable

14. Did nursing staff actively facilitate communication between surgeons and family members when needed?

(1) Never (2) Seldom (3) Sometimes (4) Frequently (5) Always (6) Not applicable

15. Were your feedback/suggestions regarding care services attentively received by nursing staff?

(1) Never (2) Seldom (3) Sometimes (4) Frequently (5) Always (6) Not applicable

**Information Support**

16. During postoperative recovery, did nursing staff explain post-procedure protocols and next steps?

(1) Strongly Disagree (2) Disagree (3) Neutral (4) Agree (5) Strongly Agree

17. During recovery, were you informed about your vital signs and health status?

(1) Strongly Disagree (2) Disagree (3) Neutral (4) Agree (5) Strongly Agree

18. During postoperative follow-up, did nursing staff provide tailored postoperative care instructions?

(1) Strongly Disagree (2) Disagree (3) Neutral (4) Agree (5) Strongly Agree
